# Effect of prolonged freezing of semen on exosome recovery and biologic activity

**DOI:** 10.1038/srep45034

**Published:** 2017-03-24

**Authors:** Jennifer L. Welch, Marisa N. Madison, Joseph B. Margolick, Shannon Galvin, Phalguni Gupta, Otoniel Martínez-Maza, Chandravanu Dash, Chioma M. Okeoma

**Affiliations:** 1 Department of Microbiology and Immunology, Carver College of Medicine, University of Iowa, 51 Newton Road, Iowa City, IA, 52242-1109, USA; 2 Miami Dade College, Homestead Campus, 500 College Terrace, Homestead, FL, 33030, USA; 3 Department of Molecular Microbiology and Immunology, John Hopkins Bloomberg School of Public Health, 615 N. Wolfe Street, Baltimore, MD, 21205, USA; 4 Department of Medicine and Infectious diseases, Northwestern University Feinberg School of Medicine, 645 N Michigan Avenue, Chicago, IL, 60611, USA; 5 Department of Infectious Diseases and Microbiology, University of Pittsburgh, 426 PUBHL130 DeSoto Street, Pittsburgh, PA, 15261, USA; 6 UCLA AIDS Institute, University of California, Los Angeles, 615 Charles E. Young Drive South, Los Angeles, CA, 90095, USA; 7 Center for AIDS Health Disparities Research, Department of Biochemistry and cancer biology, Meharry Medical College,Nashville, TN 37208, USA; 8 Interdisciplinary Graduate program in Molecular and Cellular Biology (MCB), University of Iowa, Iowa City, IA, 52242, USA

## Abstract

Exosomes are important vehicles of intercellular communication that shape host responses to physiologic, tumorigenic, and pathogenic conditions. The composition and function of exosomes are dynamic and depends on the state and condition of the cellular source. In prior work, we found that semen exosomes (SE) from healthy donors who do not use illicit drugs potently inhibit HIV-1. Following semen donation, specimens are either used immediately or frozen for use at a later time. It has been shown that short-term freezing of semen has no effect on SE-mediated HIV-1 inhibition. However, the effect of illicit drugs and prolonged freezing on SE bioactivity is unknown. Here, we show preservation of SE physical properties, (morphology, concentration, intensity/size) irrespective of illicit drug use or duration of semen freezing. Interestingly, illicit drugs and prolonged freezing decreased the levels of SE-bound CD63/CD9 and acetylcholinesterase activity respectively. Furthermore, we show differential effects of illicit drug use and prolonged freezing on SE-mediated HIV-1 inhibition. Our results highlight the importance of the source of SE and condition of semen storage on SE content and function. In-depth evaluation of donor drug-use and duration of semen storage on SE cargo and bioactivity will advance our understanding of SE composition and function.

Exosomes are nano vesicles secreted by various cell types into the extracellular milieu, including semen. Semen exosomes (SE) enwrap various RNA (micro RNA [miRNA] and messenger RNA [mRNA]) and protein cargos^1^,^2^,^3^,^4^. Exosomal proteins such as tetraspanins (CD9, CD63, and CD81) are commonly used as markers of exosome^1^,^5^. The protein and RNA content of SE have the potential to be used as intercellular messengers, biomarkers, or therapeutic tools for reproductive disorders and sexually transmitted diseases^6^. Indeed, previous studies have found that SE contain inhibitory molecules that restrict retroviral infection, including infection with MLV and HIV-1 in cultured cells^1^,^2^ and in a mouse model of retroviral infection^2^.

SE are commonly purified from semen of donors who provide samples for reproductive purposes or for disease and drug screening. In most cases, semen samples not immediately used for isolation of spermatozoa or for other downstream analyses are placed in frozen storage, typically at −80 °C. Consequently, prolonged storage of semen in repositories or biobanks is possible. Such repositories serve as sources for semen samples that may be used in retrospective studies that address specific biological, functional, or medical questions. Moreover, conducting prospective studies involving multiple experimental time points that must be analyzed together requires freezing semen samples as they are collected. In clinical and laboratory settings, samples collected on the weekend or outside normal business hours are routinely stored frozen (possibly in −80 °C) for further processing. Thus, retrospective and prospective studies will use frozen semen samples for studies requiring SE.

While short-time freezing of semen does not affect SE function such as anti-HIV activity^1^,^2^, the effect of prolonged freezing on SE recovery, and biological activity is unknown. Furthermore, the effect of donor illicit drug use on SE characteristics is not known. Here, we made unexpected but novel observations on the differential effects of illicit drugs and prolonged freezing of semen on SE protein content and SE-mediated inhibition of HIV-1. The findings have identified potential markers of interest and have provided new insights regarding how exposure to illicit drugs and environmental conditions may impact SE cargo composition and biological activity.

## Results

### Effect of prolonged freezing of semen on SE protein concentration

Donor and semen characteristics are presented in Table 1. Following isolation, we examined SE concentration by measuring the protein content of intact SE using Bradford assay^1^,^2^. We found that SE concentration ranged between 5.55 μg/μl to 6.37 μg/μl. The protein content of intact SE is independent of length of storage and donor drug use. These data indicate that SE recovery was similar in the short and prolonged samples (Table 2).

### Prolonged freezing of semen and SE physical properties

**Figure 1:**
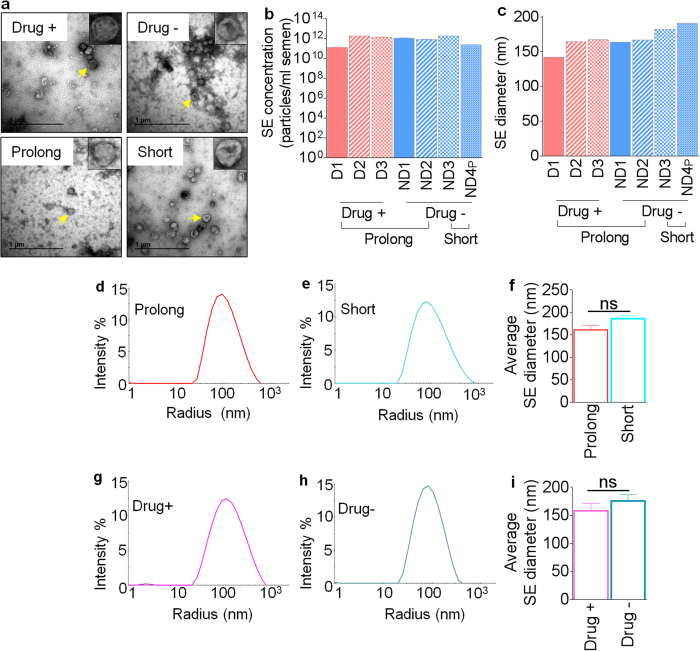
Physical properties of SE isolated from diverse conditions. (**a**) Representative TEM images showing SE morphology from donors who used illicit drugs (top left) or did not use illicit drugs (top right) and following prolonged (bottom left) or short (bottom right) term freezing of semen. The insets on each image show the zoomed image of a single vesicle. Yellow arrowheads indicate the vesicle in each field used for the zoomed image. Scale bars are 1 μm. (**b**) Exosome concentrations were quantified by NTA and averaged from three measurements. Concentrations were calculated per ml of semen. (**c**) Comparison of SE size in diameter (nm) by length of storage or donor illicit drug use. (**d**,**e**) Representative histograms showing SE distribution intensity by radius (nm) following prolonged (n = 5) or short-term freezing (n = 13) of semen. (**f**) Average SE size in diameter (nm) by length of storage. (**g**,**h**) Representative histograms showing distribution intensity of SE from donors who used (n = 3) illicit drugs or donors who did not use illicit drugs (n = 15). (**i**) Average SE size in diameter (nm) by donor illicit drug use. For statistical analysis, samples were grouped into “length of the freezing” and “drug use” and analyzed comparing short to prolong or drug− to drug+ respectively for F and I. Significance was determined by student’s t test. Differences with p values of 0.05 are considered significant. Error bars are standard errors of the mean. ns = not significant.

To analyze possible changes in physical properties of SE following prolonged freezing, SE isolated from prolonged frozen samples were examined for physical characteristics using transmission electron microscopy (TEM), NanoSight nanoparticle tracking analysis (NTA), and dynamic light scattering (DLS) measurements. Post-isolation TEM revealed similar morphology in all SE populations regardless of donor drug use or length of freezing (Fig. 1a). Evaluation of SE concentration in donor semen by NTA showed that approximately 1.28 × 10^11^–1.78 × 10^12^ SE were isolated from semen. This range sits within the expected particle concentration^4^ and revealed that length of storage and donor drug use has no significant effect on the number of SE isolated from donor semen (Fig. 1b). Additional characterization of SE intensity and size distributions were obtained using DLS. The radius of the particles was calculated using sphere approximation. Our data show that length of freezing has no effect on SE size distribution (Fig. 1c–f). Similarly, drug use by semen donors has no effect on SE size distribution (Fig. 1c,g–i). These results indicate that prolonged freezing of semen has no effect on the physical properties of SE irrespective of donor drug use status.

### Effect of prolonged freezing of semen on SE protein cargo

**Figure 2:**
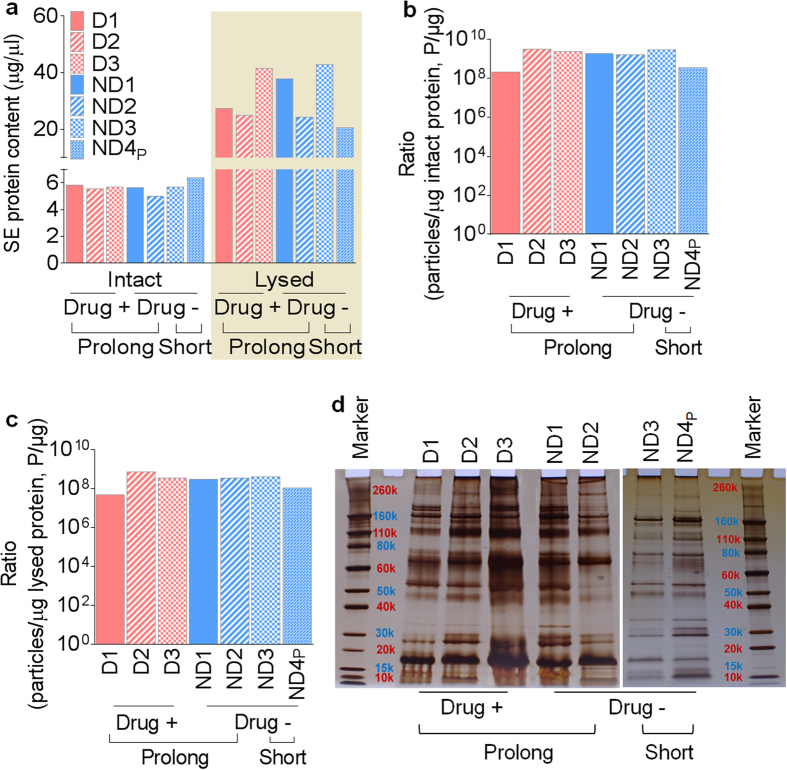
Protein concentration and electrophoretic patterns of SE from different donors at different freezing times. (**a**) Protein concentrations as determined by Bradford analyses for intact and lysed SE. (**b**) Comparison of ratio of particles to intact protein across all donor samples and under different storage conditions. (**c**) Comparison of ratio of particles to total protein across all donor samples and under different storage conditions. (**d**) SE protein footprint analyzed by silver stain.

Although prolonged freezing of semen preserves SE physical properties, the effect of prolonged freezing on SE protein composition is unknown. Thus, we assessed the effect of prolonged freezing of semen on SE protein quality and composition. SE were lysed and total protein concentration was quantified. The data show presence of protein in all SE samples, with some donor-dependent variations (Fig. 2a; see intact vs lysed bars). However, prolonged freezing of semen did not alter total SE protein, regardless of donor drug use status. Although semen exosomes are a distinctly heterogeneous population of vesicles^1^,^7^ (Fig. 1a), it is currently unknown to what extent protein concentration varies with vesicle numbers. To ascertain how SE particle number (P) relate to protein concentration (μg), we calculated the ratio of particle to protein (P/μg). We observed subtle donor-dependent differences in SE particle to protein ratios with numbers ranging from approximately 2.2 × 10^8^ to 3.0 × 10^9^ particles per μg intact protein (Fig. 2b). For completeness, we also computed the ratio of particle to total protein (4.7 × 10^7^ to 7.1 × 10^8^) and found that particle to protein ratio diminishes with increasing protein concentration (Fig. 2c). Our data suggest that SE protein content varies independent of vesicle number, donor drug use, or length of freezing.

Since prolonged freezing of semen does not decrease the protein content of SE, we sought to determine whether the protein profile will be altered following prolonged freezing. Thus, we examined SE proteome profile by performing protein foot printing. As shown in Fig. 2d, random differences in band intensity were observed in the SE protein footprint. These differences are independent of freezing length and donor drug use. There was no discernable difference in SE proteome pattern following 30 years of prolonged frozen semen storage when compared to semen stored for only 2 years. Similarly, donor illicit drug use did not change SE protein footprint (Fig. 2d).

**Figure 3:**
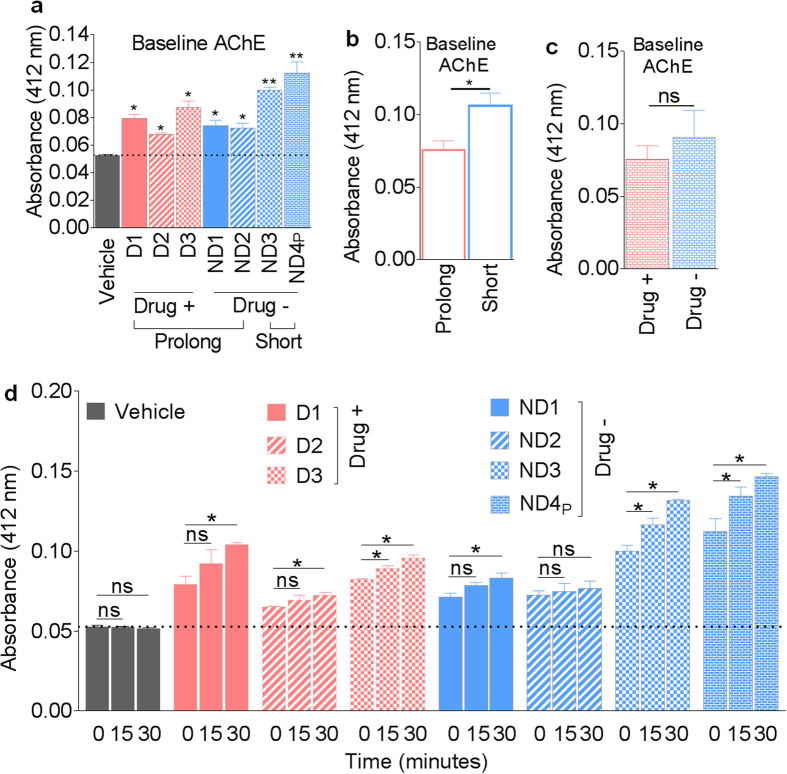
Length of semen storage alters SE-associated AChE enzymatic activity. Acetylcholine-esterase (AChE) specific enzyme activity was measured in SE as described in “Methods” where vehicle is PBS. (**a**) Baseline acetylcholine-esterase activity taken at time = 0 minutes for individual donor and pooled SE. (**b**) Average baseline AChE enzymatic activity at time = 0 for prolonged (n = 5) and short-term freezing (n = 13). (**c**) Average baseline AChE enzymatic activity for illicit drug use (n = 3) and no illicit drug use (n = 15). (**d**) Time course of AChE activity at time = 0, 15, and 30 minutes. Statistics was based on comparing baseline AChE activity to vehicle, short to prolong, and Drug− to Drug+ respectively for panels a, b, and c. For panel D, statistics time values from point 15 and 30 minutes were compared to the 0 time point. Significance was determined by student’s t test. Differences with *p* values of 0.05 or less are considered significant *p < 0.05, **p < 0.001. Error bars are SEM. ns = not significant.

To further determine if prolonged freezing of semen alters the level of specific SE protein, we evaluated the activity of acetylcholine-esterase (AChE)—an enzyme typically associated with exosomes^8^. Analysis of AChE activity shows that all SE samples contain AChE with donor-dependent variability (Fig. 3a). We found that prolonged (30 years) freezing of semen significantly decreased basal SE AChE activity (Fig. 3b) while donor drug use has no significant effect on basal AChE activity (Fig. 3c). As expected, measurement of the levels of SE AChE activity at different time points (0, 15, 30 minutes) show a time-dependent increase in AChE activity in all donor samples (Fig. 3d). These data serve to validate that the isolated vesicles are SE. The lower basal SE AChE activity seen after prolonged frozen storage (Fig. 3b) is indicative of potential AChE degradation or loss of enzymatic function following prolonged frozen semen storage.

The protein content and footprint results (Fig. 2a,d) suggest that the proteome profile of SE is not markedly altered by prolonged freezing or by donor drug use. However, levels of specific proteins or enzymatic activities may be markedly altered by prolonged freezing as revealed by the results of AChE activity (Fig. 3a–d). To further test this hypothesis, we assessed the effect of prolonged freezing and donor drug use on the level of the ubiquitous exosomal markers, CD63 and CD9^1^,^5^.

**Figure 4:**
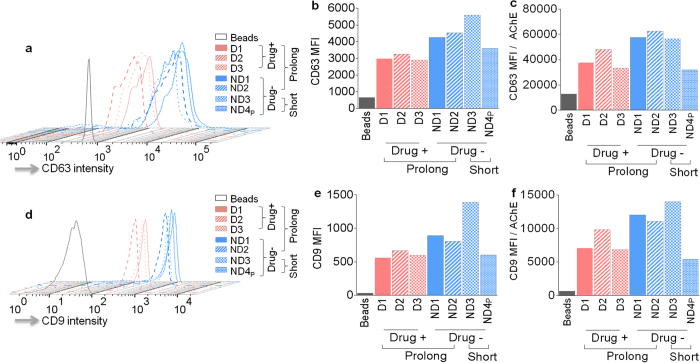
Levels of CD63 and CD9 on the surface of SE. Analysis of SE-bound CD63 and CD9 was performed as described in the “Methods” section by binding of SE to anti-CD63 coated magnetic beads. CD63-bound SE were stained with human CD63 or CD9 and analyzed by flow cytometry. (**a**) Staggered histogram showing changes in CD63 on the surface of SE. (**b**) Mean fluorescence intensity (MFI) of CD63 on the surface of SE. (**c**) CD63 MFI normalized to baseline AChE. (**d**) Staggered histogram of CD9 levels on the surface of SE. (**e**) CD9 MFI on SE. (**f**) CD9 MFI normalized to baseline AChE.

As expected, all semen samples contain CD63 and CD9 positive SE, irrespective of length of freezing (Fig. 4a–f). Strikingly, CD63 and CD9 phenotyping produced interesting distinction that separates SE based on donor drug use. SE purified from donors that used illicit drugs have reduced surface CD63 and CD9 intensity compared to SE purified from donors who did not use drugs (Fig. 4a–f). To further validate this observation, CD63 and CD9 MFI were normalized to baseline AChE activity (Figs. 4c,f). In contrast, prolonged freezing has no significant effect on CD63 and CD9 content of SE (Fig. 4a–f). These results indicate that a drug-dependent mechanism may be involved in the suppression of tetraspanin protein (CD63 and CD9) expression in the cells that secrete SE or in the incorporation of CD63 and CD9 into SE.

### Effect of prolonged freezing of semen on SE RNA content

**Figure 5:**
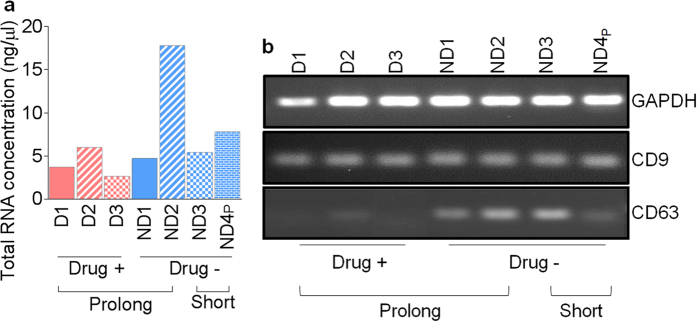
Content and quantity of SE RNA from different donors and after different freezing times. RNA was extracted from SE as described in “Methods”. (**a**) Total RNA concentration from 12 μg of SE. (**b**) RT-PCR analysis of mRNA of selected exosomal markers and GAPDH in SE. PCR products were from cDNA generated using equivalent amounts (1 ng/μl) of total RNA.

Our previous work revealed that SE isolated from semen contain substantial amounts of RNA^1^. However, whether SE RNA content change following prolonged freezing is unknown. This was assessed by isolation of total SE RNA followed by evaluation of RNA concentration by spectrophotometric analysis. We found donor-dependent variations in SE RNA content (Fig. 5a). The observed donor-dependent difference in RNA content is independent of donor drug use or prolonged freezing of semen.

Given the drug-use dependent difference in CD63 and CD9 protein content (Fig. 4a–f) and the donor-dependent difference in total RNA content (Fig. 5a), we sought to determine whether prolonged freezing and donor drug use altered the level of coding mRNA loaded into SE. Reverse transcription PCR (RT-PCR) revealed that there was no difference in the abundance of Glyceraldehyde 3-phosphate dehydrogenase (GAPDH) and CD9 mRNA (Fig. 5b). Interestingly, we observed a significant decrease in CD63 mRNA in SE from semen of donors that used illicit drugs (Fig. 5b). These data identify CD63 as a gene that is susceptible to modulation by illicit drugs both at the mRNA and protein levels. Whether the action of illicit drugs on CD63 is directed at exosome-producing cells and/or packaging into semen exosomes is currently unknown and being investigated. The reason for the discrepancy between CD9 protein and mRNA is unknown. It is possible that SE originate from various parts of the male reproductive organs and that the protein and mRNA content of SE could vary depending upon the cell from which the SE was secreted.

### The effect of prolonged freezing of semen on SE-mediated inhibition of HIV-1

It is known that SE isolated from semen that had been stored for a short time, obtained from healthy donors with no history of drug use, robustly inhibit HIV-1 infection^1^,^2^. The inhibitory effect of SE on HIV-1 infection is operative when cells are pre-treated with SE 24 hours before infection and when SE are pre-incubated with virus for 1 hour at 37 °C before addition of the SE/virus complex to cells^1^. These two different models of SE inhibition signify that SE may use multiple mechanisms to inhibit HIV-1 infection. To assess whether prolonged (30 years) freezing of semen or donor drug use alter SE-mediated inhibition of HIV-1, we evaluated SE inhibitory characteristics in both pre-treatment and pre-incubation inhibition models.

**Figure 6:**
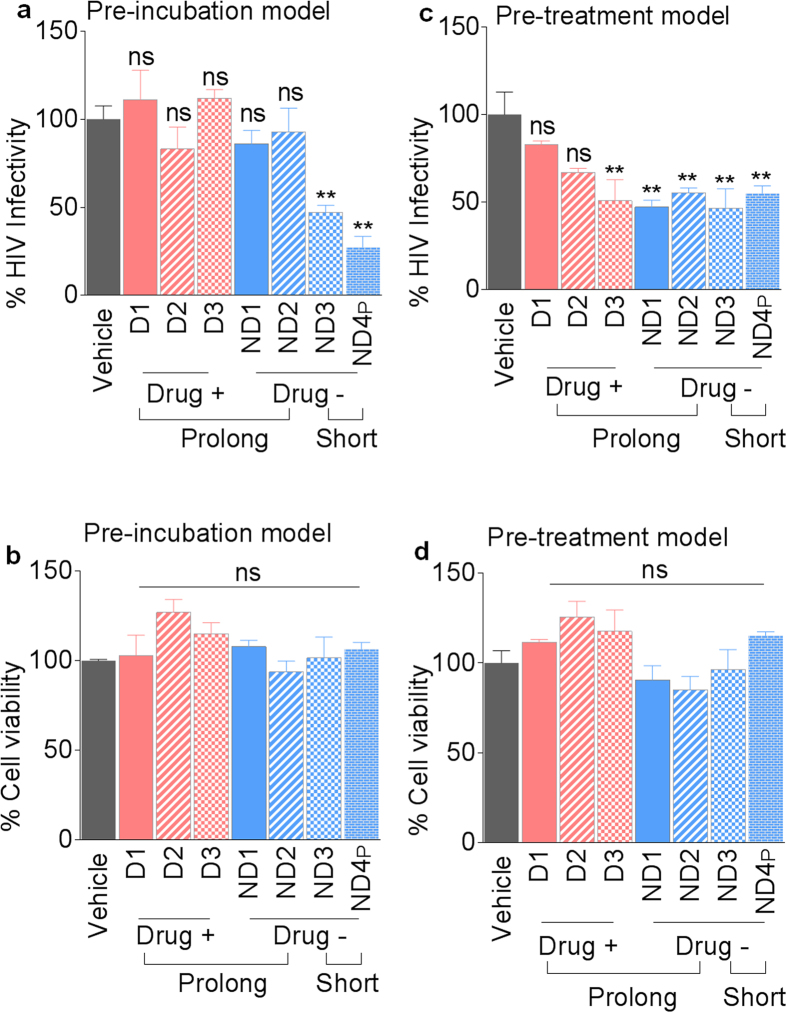
Effects of length of freezing and donor drug use on SE-mediated HIV-1 inhibition. (**a**,**b**) Infectivity of HIV-1 and cell viability in a pre-incubation infection model where SE (100 μg/ml) or vehicle PBS was pre-incubated with 8 RT units of HIV-1 NL4.3 for 1 hour at 37 °C before infection of TZM-bl indicator cells for 24 h. (**a**) Infectivity and (**b**) Cell viability. (**c**,**d**) Infectivity of HIV-1 and cell viability in a pre-treatment infection model where SE (100 μg/ml) or vehicle PBS was added to TZM-bl indicator cells for 24 h before infecting cells with 8 RT units of HIV-1 NL4.3 for 24 h. (**c**) Infectivity and (**d**) Cell viability. Vehicle is set as reference at 100% for infectivity and viability. Statistics was performed by comparing infectivity or viability values from each donor to vehicle control. Significance was determined by student’s t test. Differences with *p* values of 0.05 or less are considered significant *p < 0.05, **p < 0.001. Error bars are SD. ns = not significant.

Pre-incubation of SE with HIV-1 for 1 hour before addition of the SE/virus complex to cells reveals an impairment of SE inhibitory effect of HIV-1 infection that is mediated by prolonged freezing of semen (Fig. 6a). As expected, SE isolated from semen stored for ~2 years effectively inhibited HIV-1 infection (Fig. 6a), supporting previous reports^1^,^2^. The loss of HIV-1 inhibition by SE following prolonged freezing was not due to cell death because cell viability as determined by MTT assay was unaffected (Fig. 6b). In this SE inhibition model, donor drug use has no effect on SE-mediated HIV-1 inhibition as well as on cell viability (Fig. 6a,b; compare pink bars vs blue bars).

SE endows cells with anti-HIV phenotype as shown by marked inhibition of HIV-1 infection of cells following exposure of cells to SE prior to infection^1^. To evaluate the effect of prolonged (~30 years) freezing of semen on SE-mediated anti-HIV-1 effect on target cells in the pre-treatment condition, we incubated SE with cells for 24 hours prior to infection with HIV-1. All SE from non-drug users independent of length of frozen semen storage inhibit HIV-1 infection (Fig. 6c). In contrast, 1 out of 3 SE from illicit drug users inhibit HIV-1 infection (Fig. 6c, donor D3). As in the pre-incubation model (Fig. 6a), the differential pattern of SE inhibition in the pre-treatment model could not be ascribed to cellular toxicity because cells under all conditions were equally viable (Fig. 6d).

**Figure 7:**
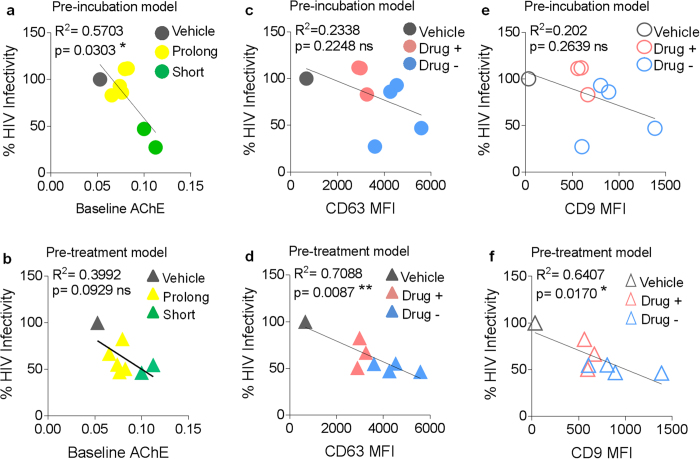
Association between CD63 expression and AChE activity with SE-mediated inhibition of HIV-1 infection. Correlation between baseline SE-AChE activity and inhibition of HIV-1 infection during (**a**) pre-incubation (**b**) pre-treatment infection models. Correlation analysis between CD63 and inhibition of HIV-1 infection during (**c**) pre-incubation and (**d**) pre-treatment infection models. Correlation analysis between CD9 and inhibition of HIV-1 infection during (**d**) pre-incubation and (**e**) pretreatment infection models. Prior to correlation coefficient analyses, normality of distribution was checked with D’Agostino and Pearson normality test. Differences with *p* values of 0.05 or less are considered significant *p < 0.05, **p < 0.01.

The AChE activity data in Fig. 3b,d and SE-mediated inhibition of HIV-1 infection in Fig. 6a suggest a link between decreased SE AChE activity and SE-mediated HIV inhibition when SE and the virus are pre-incubated before infection. Indeed, further correlation analysis by Pearson r coefficient and linear regression reveals a significant correlation between decreased SE-AChE activity and absence of SE-mediated HIV-1 inhibition in a pre-incubation infection model (Fig. 7a). In contrast, no correlation was observed between decreased AChE activity and SE-mediated HIV-1 inhibition in a pre-treatment model (Fig. 7b). These correlative analyses suggest that the AChE activity of SE or other enzymes such as butyrylcholinesterase (BChE) that is capable of hydrolyzing acetylcholine may play a role in the SE-virus inhibitory interaction in the pre-incubation model of HIV-1 infection.

In a pre-incubation SE inhibition model, no significant correlation was observed between SE-CD63 or SE-CD9 content and inhibition of HIV-1 (Fig. 7c,e, respectively). However, a significant inverse correlation was observed between surface CD63 and CD9 in SE and SE-mediated inhibition of HIV-1 infection in a pre-treatment infection model, where increased SE-CD63 or SE-CD9 content is associated with decreased HIV-1 infectivity (Fig. 7d, f respectively). This observation is in agreement with our data that show that SE with decreased CD63 or CD9 isolated from donors who use illicit drugs (Fig. 4a–d) had no effect on HIV-1 inhibition in 2 out of 3 donors (Fig. 6c). Together, these data imply that prolonged freezing rather than illicit drugs may alter components of SE that mediate a potential SE/HIV-1 interaction that occurs upon incubation of SE and HIV-1. Furthermore, our data suggest that the use of illicit drugs rather than prolonged freezing impairs SE-mediated endowment of an anti-HIV-1 state to recipient cells. This effect may depend on other donor intrinsic factors.

## Discussion

In this study we have shown that prolonged semen freezing has no significant effect on the recovery of semen exosomes (SE). Isolated SE have similar physical properties as determined by comparative morphology, light scattering patterns (intensity and size), and particle concentrations following prolonged and short-term semen freezing. We also show that donor drug use has no significant effect on SE recovery and SE physical properties. In our study, we isolated approximately 1.28 × 10^11^–1.78 × 10^12^ particles per ml of semen. This concentration is in line with previously reported SE concentration (4.7 × 10^11^–3.12 × 10^13^)^4^. Similar to our findings, others have reported minimal loss of urinary exosome-associated proteins from urine that was stored at −80 °C, however this study addressed the effect of temperature storage on exosome-associated protein recovery, and did not address length of storage^9^.

Although SE physical properties, RNA and protein contents are stable following prolonged freezing, the levels of specific SE cargos may be altered, as exemplified by decreased AChE activity (Fig. 3a–d). Such alteration may affect some biological and functional activities of SE, although this is yet to be determined. While total protein concentration (intact and lysed) and protein footprint is conserved in all SE samples, prolonged semen freezing reduced the level of AChE activity of SE. There was also a trend towards drug-induced reduction of SE AChE activity, but this difference did not reach statistical significance. AChE is a plasma membrane protein incorporated into exosomes during exosome biogenesis, making the enzymatic activity of AChE a commonly used exosome marker^10^. AChE activity is indicative of the enzymatic degradation of acetylcholine, a compound known to dampen inflammatory response in cells^11^. Inhibition of AChE with pyridostigmine downregulates HIV-1-induced T cell activation and T cell proliferation in chronically infected HIV-1 patients, however, viral load was not assessed in this study^11^. While a link between AChE activity and HIV-1 viral load has not been made, infection with herpes simplex virus type 1 (HSV-1) results in a steady decline in the enzymatic activity of AChE^12^, indicating that AChE or BChE may play a role in viral infection. Thus, decreased AChE activity in SE following prolonged storage of frozen semen may have important biological or functional effects. Indeed, the correlation (Fig. 7a) between decreased AChE activity (Fig. 3b) and loss of HIV-1 inhibition by SE following prolonged freezing (Fig. 6a) suggest that prolonged freezing of semen may concurrently or independently alter the activities of proteins that inhibit HIV-1 infection. Future studies are needed to investigate the role of SE-bound AChE, as well as other SE-bound proteins/enzymes in modifying processes that inhibit HIV-1 infection, and SE isolated from semen stored for at least 30 years will be valuable for such studies.

In contrast to AChE levels, prolonged freezing of semen has no effect on SE CD63 and CD9 protein and mRNA. However, drug use by semen donors markedly reduced the level of CD9 protein, as well as CD63 protein and mRNA. CD63 and CD9 are cellular membrane proteins found in exosomes including SE^1^. It has been shown that CD63, CD9 and other tetraspanin proteins play multiple important roles in HIV-1 infection^13^,^14^,^15^,^16^ and that CD63 and CD9 are incorporated into released HIV-1 particles^15^,^17^. Inhibition of CD63 reduces HIV-1 infection in macrophages^14^ in a CCR5 co-receptor dependent manner^14^. On the contrary, CD63 protein incorporated into released virions attenuates HIV-1 infectivity in a virion-specific manner^15^, and recombinant extracellular domains of CD63 potently inhibit HIV-1 infection in a cell type-dependent manner^13^. These reports suggest that the effect of CD63 on HIV-1 may depend both on target cells and on the virions. Although the role of CD9 in HIV infection has not been extensively studied, it has been shown that overexpression of CD9 reduced HIV-1 infectivity^16^. Additionally, CD9 has been implicated in regulating virion membrane fusion events where knock-down of CD9 expression enhanced viral entry^18^. Our finding that donor drug use reduces CD63 protein and mRNA, as well as CD9 protein (Figs 4 and 5) and that such SE containing lower levels of CD63 and CD9 were unable to inhibit HIV-1 infection in a pre-treatment SE inhibition model (Fig. 6c) suggest that CD63 and/or CD9 protein in SE may be linked to SE-mediated inhibition of HIV-1 in this model. The amount of CD63 and CD9 at the surface of SE clearly correlates with the level of HIV-1 inhibition in a pre-treatment model (Fig. 7d,f) and inversely correlates with donor drug use. Further studies will be needed to link SE CD63 and CD9 to HIV-1 inhibition and to define the role of illicit drugs in the process.

Similar to prolonged freezing of semen, donor drug use may alter SE cargo composition and function, such as SE-mediated inhibition of HIV-1 infection^1^,^2^. It is known that addictive stimulant drugs including cocaine increase the risk of exposure to HIV-1 infection and investigation into the chronic administration of cocaine and HIV-1 infection showed significant enhancement of cell death and toxic effects^19^. Therefore, it is probable that illicit drug use alters the composition of exosomes during biogenesis due to its cellular affects. The impact of drug use on the exosomal pathway is further exemplified by the observation that cocaine enhanced the release of extracellular vesicles in cell cultures^20^. Indeed, the effect of illicit drugs goes beyond enhancement of HIV-1 infection. Illicit drugs such as, opioid narcotics, methamphetamines, marijuana, and cocaine have been found to adversely impact male fertility^21^, although their mechanisms of action are currently unknown. Thus, identifying proteomic differences in semen exosomes isolated from donors who reported illicit drug use compared to non-user and their resulting effects on HIV-1 infectivity or male fertility may be essential in identifying exosome-induced recipient cell protective effects against HIV-1 or detrimental effects on reproduction.

The fundamental roles of SE in HIV-1 inhibition are intriguing. However, the mechanisms of action and the inhibitory factors need to be identified. The observed distinctive effects of illicit drug use and prolonged freezing of semen on the different SE inhibition models (pre-incubation vs pre-treatment) support the proposition that SE may utilize different mechanisms or pathways to inhibit HIV-1 infection. Thus, SE isolated from semen of illicit drug users and semen that has been frozen at −80 °C for a prolonged time may be used in elucidating the mechanisms of SE anti-HIV-1 activity and in identifying the inhibitory factors in SE. Additionally, SE from semen of illicit drug users may serve as a useful tool for identifying predictive bio-signatures in cases of chronic drug exposure.

Finally, although the findings of this study are intriguing, our study is not free from limitations. The small sample size limits our conclusions and requires caution in interpreting the observed associations. Increased sample size is needed to overcome these constraints and to make conclusive statements in terms of the i) link between SE AChE, CD63, and CD9 to HIV-1 inhibition and ii) causation of the phenotypes observed in SE.

## Methods

### Ethics statement

This study involves the use of existing human specimens (semen) and therefore is not human subjects’ research. Dr. Amy E.T. Sparks, Director of University of Iowa In Vitro Fertilization and Reproductive Testing Laboratory provided de-identified samples of human semen from healthy donors. These samples were discarded from routine examinations and not linked to any identifiers. In addition, the Multicenter AIDS Cohort Study (MACS) provided de-identified semen samples from donors who at the time of collection reported using or not using illicit drugs. The MACS samples are also not linked to any identifiers. This study was approved by the University of Iowa Institutional Review Board.

### Semen donors

A total of 18 donors were used for isolation of exosomes from semen in this study. Five donor semen samples were received frozen from the Multicenter AIDS Cohort Study (MACS). The MACS samples were collected approximately 30 years ago (1986) and stored in −80 °C until used. As control, 13 semen samples (one independent sample and 12 independent samples that were pooled into one sample) from the University of Iowa In Vitro Fertilization and Reproductive Testing laboratories were used. The University of Iowa samples were collected in 2014 and stored in −80 °C until used. All semen samples were thawed at room temperature. SE were isolated using a protocol previously described^1^,^2^ from semen of various donors who, at the time of collection, were using or not using cocaine or other illicit drugs. The rationale for inclusion of the two donor archetypes is to gain insight into how illicit drugs affect SE recovery and function. Semen samples were frozen at −80 °C since collection (~30 years ago) until thawed for this study, and will be referred to as prolonged freezing. These samples were collected and stored by the Multicenter AIDS Cohort Study (MACS), which provided these specimens for the current study. To determine the effect of prolonged freezing of semen on the recovery of SE, we used SE isolated from semen samples obtained from the University of Iowa and stored for about two years under similar conditions, referred to as short-term freezing as control. All samples are from donors with no history of human immunodeficiency virus (HIV), hepatitis B virus (HBV), and hepatitis C virus (HCV) infection.

### Cells and viruses

TZM-bl cells were obtained through the NIH AIDS Reagent Program and maintained in DMEM (Gibco-BRL/Life Technologies) complete with 5% exosome-depleted FBS (Gibco), 100 U/ml penicillin, 100 μg/ml streptomycin, sodium pyruvate and 0.3 mg/ml L-glutamine (Invitrogen, Molecular Probes) as previously described^1^. HIV-1_pNL4.3_ was obtained from the NIH AIDS Reagent Program and transfected into HEK293 cells using Lipofectamine 2000 according to the manufacturer’s instructions (Invitrogen) and as previously described^1^. HIV-1 was purified from cell culture supernatant and clarified by centrifugation and 0.45 μm filtered. Viral titer was determined by EnzChek Reverse Transcriptase Assay (Life Technologies)^1^.

### Isolation of semen exosomes (SE)

Semen was thawed at room temperature to allow for liquefaction. To pellet spermatozoa/cellular debris, samples were centrifuged at 7000 × g for 30 min at 4 °C. Cell-free seminal plasma was placed in a fresh conical tube and ExoQuick reagent (SBI) was added at a ratio of 4:1. Samples were re-suspended by inversion before incubation at 4 °C overnight without rotation per manufacturer’s instructions. The seminal plasma/ExoQuick mixture was centrifuged at 1500 × g for 30 min at 4 °C after which the exosome-free supernatant was removed from the exosome pellet. To remove residual ExoQuick, the exosome pellet was centrifuged at 1500 × g for 5 min. Any remaining supernatant was discarded from the exosome pellet, and the pellet was re-suspended in 1 × PBS in 1/5 of the original semen volume. This pellet is the semen exosome herein referred to as SE. SE were quantified by Bradford assay and aliquoted before use. To isolate SE from pooled donors, cell-free seminal plasma from 12 donors was combined in a conical tube before additional of ExoQuick reagent after which the protocol was followed as described above.

### Transmission electron microscopy

50 μg of SE were resuspended in 2.5% glutaraldehyde fixation solution and incubated at 4 °C for 1 hr. After which, the fixed exosomes were ultracentrifuged at 100,000 × *g* for 2 hr at 4 °C to pellet the vesicles and remove the fixation solution. The fixed vesicle pellets were resuspended in PBS and deposited on Formvar film with carbon-coated 400-copper grids. The samples then underwent negative staining with 1% uranyl acetate for 1 min and were allowed to air-dry. Images were acquired and viewed using JEOL JEM 1230 transmission electron microscope (TEM).

### Quantification of SE concentration

NanoSight LM10 nanoparticle tracking analysis was used to determine concentration of SE particles per ml of semen and for estimation of particle numbers per μg of protein. Exosome preparations from each sample were diluted in PBS to a final volume of 0.5 ml and were assayed in triplicate. NanoSight NTA software was used to analyze average particle concentration from the three replicates.

### Quantification of SE protein (intact and lysed) content

Intact SE protein quantification was determined by Bradford assay and absorbance at 595 nm. Total concentration of isolated SE was computed by multiplying the SE concentration in μg/μl by the volume of re-suspended SE. To quantify SE total protein which includes the luminal protein content, 5 μg of SE were lysed in 0.1 M NaCO_3_ for 1 hr at 4 °C before protein quantification by Bradford assay.

### Protein foot-printing

5 μg of SE from each donor were lysed and separated on a 4–12% polyacrylamide gel before silver staining using the Pierce Silver Stain kit (Thermo Scientific) per the manufacturer’s instructions.

### Dynamic light scattering of SE

A concentration of 0.1 mg/ml of SE in a 150 μl volume of PBS were analyzed by DynaPro NanoStar DLS (Wyatt Technologies) using disposable UVettes (Eppendorf). 10 measurements per SE sample were completed at a constant temperature of 25 °C and laser wavelength of 665 nm. Analysis was determined by an average of ten measurements per SE sample. Data were analyzed using Dynamics software.

### Acetylcholinesterase (AChE) activity

Acetylcholinesterase is an exosome specific enzyme^8^ commonly used to identify exosomes and to differentiate exosomes from viruses. 50 μg of SE were lysed in a 1:1 volumetric ratio in 2% Triton X-100 in PBS. For negative control, equivalent volume of PBS was used. 5 μl of SE/Triton X fraction was added to a 96 well flat-bottom clear plate in triplicate. 1.25 mM acetylthiocholine chloride (AChE) (Sigma-Aldrich) and 0.1 mM 5,5′-Dithiobis 2-nitrobenzoic acid (DNTB) (Sigma-Aldrich) were added in a final volume of 100 μl to the exosome containing wells. Kinetic absorbance was read at 450 nm for 30 min at 37 °C at 5-minute time intervals.

### Quantification of CD63 and CD9 content of SE

Purification of SE was further verified by expression of the exosome markers, CD63 and CD9^1^,^22^. Detection of human CD63 and human CD9 in SE using flow cytometry was completed per exosome-human CD63 isolation/detection kit following manufacturer’s instructions (Invitrogen). Briefly, 25 μg of SE re-suspended in isolation buffer (0.1% BSA in PBS) were incubated with 20 μl of 4.5 μm-diameter magnetic polystyrene beads (Dynabeads) at 4 °C overnight with rotation. The Dynabeads are pre-coated by the manufacturer with a primary monoclonal antibody for human CD63 antigen. The SE-bound beads were then washed three times (0.1% BSA in PBS) to remove unbound exosomes, and the exosome-bound beads were stained for flow cytometry with anti-CD63-FITC (Biolegend) or anti-CD9-PE (Biolegend) for 1 hr at RT in the dark on an oscillating mixer. The SE-bound beads were then washed three times (0.1% BSA in PBS) to remove unbound antibody. CD63 or CD9 levels were analyzed through a FACSVerse instrument and mean fluorescence intensity was determined using FlowJo software (Tree Star). Further details are provided in the Supplementary Material in accordance with MIFlowCyt standards^23^.

### RNA evaluation

Total SE RNA was extracted from 12 μg of SE per donor. SE RNA was purified using RNEasy kit per manufacturer’s instructions (Qiagen). RNA was subjected to treatment with DNase (Qiagen) as previously described^1^. RNA purity was assessed by the ratio of RNA absorbance (A_260_/A_280_). An A_260_/A_280_ ratio of >2.0 signifies a pure RNA preparation^24^. RNA concentration was determined by NanoDrop RNA absorbance, and equivalent concentration of RNA (1 ng/μl) was used for cDNA synthesis (ABI). Gene specific primers were used to amplify human CD9, CD63, and GAPDH (internal control) using ABI 7500 Fast real-time PCR System as previously described^1^. Thermocycler conditions were as follows: initial denaturation at 95 °C for 1 min; 45 cycles of 10 sec denaturation at 95 °C, and 30 sec annealing at 60 °C. PCR amplicons were separated and visualized on a 2% agarose gel stained with 50 μg/ml of ethidium bromide under UV light. Images were acquired with -UVP GelDock-It imaging system (UVP).

### SE inhibition assay

The effectiveness of SE at inhibiting HIV-1 infection was accessed using two different SE inhibition (pre-incubation and pre-treatment) models as previously described^1^. In the pre-incubation model, SE was pre-incubated with HIV-1 prior to addition to target cells. This model mimics the events in HIV-1-infected men where SE is in contact with semen and SE-containing semen is discharged into the mucosa. Hence, 100 μg/ml SE or equivalent volume of vehicle (PBS) were added to 8 RT units of HIV-1_NL4.3_. The mixtures were incubated at 37 °C for 1 hour in complete DMEM containing 5% exosome-free FBS. After 1 hour, the SE/HIV-1 or HIV-1/PBS mixture was added to TZM-bl cells. Infectivity was determined 24 hours later by Steady-Glo luciferase substrate (Promega) emission in a luminometer. On the other hand, in pre-treatment model, SE are added to target cells to allow SE to condition the target cells prior to infection with HIV-1. This model assumes that SE elicits or endows anti-HIV-1 response in recipient cells. Thus, 100 μg/ml SE or vehicle (PBS) were added to equivalent number of TZM-bl cells and incubated for 24 hours. Then 8 RT units of HIV-1_NL4.3_ was added to cells. Infectivity was assessed 24 hours after the addition of virus by Steady-Glo emission. Experiments were repeated in duplicate with replicates of three per experiment.

### Cell viability

The effect of SE on HIV-1 infected cells was assessed in both pre-incubation and pre-treatment SE inhibition models. Viability was determined by the MTT assay, as previously described^25^ with replicates of three per experiment. Briefly, HIV-1 infected cells +/− SE or vehicle (PBS) and uninfected controls were incubated with 5 mg/ml MTT reagent for 3 hr in the dark at 37 °C. Following, MTT solvent (0.1% NP-40 and 4 mM HCl in isopropanol) was added and incubated for 15 min with rocking. Absorbance was read at 590 nm using Tecan Infinite M200 Pro microplate reader.

### Statistics

Statistical analysis was performed using the GraphPad Prism 7 software. Analysis of linear regression and the Pearson correlation coefficient (r) was used to determine correlation between variables using 95% confidence intervals and two-tailed p values. Infectivity was determined in two models of infection, referred to as preincubation and pretreatment models. Linear regression and correlation analysis was performed for each model of infectivity with CD63 or CD9 surface expression or baseline AChE activity. Prior to analysis of correlation coefficients, normality of distribution was checked with D’Agostino and Pearson normality test. Two-tailed t test p-value (GraphPad Prism) calculations determined statistical significance; where p < 0.05 = *, p < 0.01 = **, ns = not significant. Error bars represent standard deviation (SD).

## Additional Information

**How to cite this article**: Welch, J. L. *et al*. Effect of prolonged freezing of semen on exosome recovery and biologic activity. *Sci. Rep.* **7**, 45034; doi: 10.1038/srep45034 (2017).

**Publisher's note:** Springer Nature remains neutral with regard to jurisdictional claims in published maps and institutional affiliations.

## Supplementary Material

Supplementary Data

## Figures and Tables

**Table 1:** Characteristics of donor and semen samples.

Sample ID	Semen volume received	Length of Storage (years)	Drug Use	Co	Ma	Po	PCP	EC	He	Do	Et	Al
D1	300 μl	30	Yes	+	+	+	−	−	−	−	−	mod-heavy
D2	750 μl	30	Yes	+	+	+	−	+	−	−	+	low-mod
D3	700 μl	30	Yes	+	+	−	−	+	−	−	−	binge
ND1	450 μl	30	No	−	−	−	−	−	−	−	−	low-mod
ND2	900 μl	30	No	−	−	−	−	−	−	−	−	low-mod
ND3	1000 μl	2	No	−	−	−	−	−	−	−	−	low-mod
ND4_P_	24 ml	2	No	−	−	−	−	−	−	−	−	low-mod

Co = Cocaine; Ma = Marijuana; Po = Poppers; PCP = Phencyclidine; EC = Ecstasy; He = Heroin; Do = Downers; Et = Ethyl chloride; Al = Alcohol; + = Self-reported ever used; − = Self-reported never used; mod = Moderate; P = pooled samples from 12 donors.

**Table 2:** Effect of length of storage and illicit drug use on semen exosome protein concentration.

Sample ID	Length of Storage (years)	Storage definition	Drug Use	Intact exosome protein concentration (μg/μl)
D1	30	Prolonged	Yes	5.84
D2	30	Prolonged	Yes	5.55
D3	30	Prolonged	Yes	5.68
ND1	30	Prolonged	No	5.63
ND2	30	Prolonged	No	4.99
ND3	2	Short-term	No	5.68
ND4_P_	2	Short-term	No	6.37
