# Supplementary material for: Effect of prolonged freezing of semen on exosome recovery and biologic activity
Source: Sci Rep. 2017 Mar 24;7:45034. doi: 10.1038/srep45034 (PMC5364471; doi:10.1038/srep45034)
Supplement: Supplementary Data [file srep45034-s1.doc]

**Effect of prolonged freezing of semen on exosome recovery and biologic activity**

Jennifer L. Welch^1^
Email: [jennifer-welch@uiowa.edu](mailto:jennifer-welch@uiowa.edu)

Marisa N. Madison^1, 3^

Email: [MMadison@mdc.edu](mailto:MMadison@mdc.edu)

Joseph B. Margolick^4, 8^

Email: [jmargol1@jhu.edu](mailto:jmargol1@jhu.edu)

Shannon Galvin^5, 8^

Email: [s-galvin@northwestern.edu](mailto:s-galvin@northwestern.edu)

Phalguni Gupta^6, 8^

Email: [pgupta1@pitt.edu](mailto:pgupta1@pitt.edu)

Otoniel Martínez-Maza^7, 8^

Email: [omartinez@mednet.ucla.edu](mailto:omartinez@mednet.ucla.edu)

Chandravanu Dash^9^

Email: [cdash@mmc.edu](mailto:cdash@mmc.edu)

Chioma M Okeoma^*1, 2^
^*^Corresponding author
Email: [chioma-okeoma@uiowa.edu](mailto:chioma-okeoma@uiowa.edu)

^1^Department of Microbiology and Immunology, Carver College of Medicine, University of Iowa, 51 Newton Road, Iowa City, IA 52242-1109, USA

^2^Interdisciplinary Graduate program in Molecular and Cellular Biology (MCB), University of Iowa, Iowa City, IA, 52242, USA

^3^Miami Dade College, Homestead Campus, 500 College Terrace, Homestead FL, 33030, USA

^4^Department of Molecular Microbiology and Immunology, John Hopkins Bloomberg School of Public Health, 615 N. Wolfe Street, Baltimore, MD, 21205, USA

^5^Department of Medicine and Infectious diseases, Northwestern University Feinberg School of Medicine, 645 N Michigan Avenue, Chicago, IL, 60611, USA

^6^Department of Infectious Diseases and Microbiology, University of Pittsburgh, 426 PUBHL130 DeSoto Street, Pittsburgh, PA, 15261, USA

^7^UCLA AIDS Institute, University of California, Los Angeles, 615 Charles E. Young Drive South, Los Angeles, CA, 90095, USA

^8^Multicenter AIDS Cohort Study (MACS)

^9^Meharry Medical College

Supplementary Data

MIFlowCyt standard information for submitted flow cytometry experiments.

1. **Experiment overview**

**1.1 Purpose:**

The purpose of the experiment was to detect CD63 and CD9 expression on the surface of exosomes isolated from human semen. Exosomes were isolated from semen that had been stored for a long vs. short period and from donors that used drugs vs. nonusers. We hypothesized that all exosome samples would express both CD63 and CD9 on their surface as these are common exosomal markers.

**1.2 Keywords:** semen, exosomes, biomarkers, biofluids

**1.3 Organization:**

Okeoma Lab

Department of Microbiology

University of Iowa

51 Newton Road

Bowen Science Building, Room 3-615A

Iowa City, IA 52242

**1.4 Primary Contact:**

P.I. Dr. Chioma Okeoma [chioma-okeoma@uiowa.edu](mailto:chioma-okeoma@uiowa.edu)

Graduate Student Jennifer Welch [jennifer-welch@uiowa.edu](mailto:jennifer-welch@uiowa.edu)

**1.5 Date:**

Experiments were completed and analyzed form April-November 2016.

**1.6 Conclusions:**

Please refer to Results section in the manuscript.

**1.7 Quality Control Measures:**

PBS control was used for detection of each marker by incubating with CD63 coated magnetic beads and subsequent staining for CD63 and CD9. Stain controls were set up by staining CD63 coated magnetic beads with antibody to CD63 and antibody to CD9. Unstained magnetic beads were an additional control.

1. **Flow Sample/Specimen Description**

**2.1 Specimen Material**

**2.1.1 Biological sample:**

**2.1.1.1 Biological Sample Name**

Semen exosomes

**2.1.1.2 Biological Sample Source:**

Human semen obtained after 30 years of storage in the Multicenter AIDS Cohort Study (MACS) repository or after 2 years of storage supplied by the University of Iowa In Vitro Fertilization and Reproductive Testing laboratories.

**2.1.1.3 Biological Sample Source Organism:**

**2.1.1.3.1 Taxonomy:**

Homo sapiens

**2.1.1.3.2 Age:**

Unknown

**2.1.1.3.3 Gender:**

Male

**2.1.1.3.4 Phenotype:**

Healthy (HIV-, HBV-,HCV-)

**2.1.1.3.5 Genotype:**

Not applicable

**2.1.1.3.6 Treatment:**

Exosomes were isolated from semen using ExoQuick. After isolation, exosomes were resuspended in PBS

**2.1.2 Environmental Samples:**

Not applicable

**2.1.3 Other Samples:**

Not applicable

**2.2 Control Sample Description:**

Specificity of detection of markers was set up by incubating CD63 coated magnetic beads with PBS and staining with staining antibody for CD63 and staining antibody for CD9. Staining controls were setup by incubating each staining antibody with CD63 coated magnetic beads. Unstained magnetic beads were an additional control.

**2.3** **Sample Treatment Description:**

Exosomes were incubated with CD63 coated magnetic beads overnight. Exosomes were then washed three times and individually stained for flow cytometry with anti-CD63-FITC (Biolegend) and anti-CD9-PE (Biolegend). Staining was carried out for 1 hour at room temperature on an oscillating mixer. Exosomes were then washed three times before acquisition with FACSVerse (BD) and analysis with FlowJo (TreeStar).

**2.4 Fluorescent Reagent Description:**

| Fluorochrome | Characteristic Being Measured | Antibody Name  *Clone Name* | Vendor, cat #  *Dilution used* |
| --- | --- | --- | --- |
| FITC | Surface protein CD63 | CD63  *H5C6* | Biolegend, 353005  *1:20* |
| PE | Surface protein CD9 | CD9  *HI9a* | Biolegend, 312105  *1:20* |

**3. Instrument Details:**

**3.1 Manufacturer:** BD Biosciences

**3.2 Model:** FACSVerse Flow Cytometer System

**3.3 Instrument Configuration and Settings:**

3.3.1 Flow Fluids: Stainless steel, cuvette

Medium Flow: 60 μl/min. 10,000 events were collected for each sample.

3.3.2 Light Sources

Lasers: Blue laser, 488nm, 20mW, beam spot size 9μm x 63μm

Red laser, 640nm, 40mW, beam spot size 9μm x 63μm

Violet laser, 405nm, 40mW, beam spot size 9μm x 63μm

**3.4 Emission Detection:**

FITC 527/32

PE 586/42

PerCp 700/54

APC 660/10

SSC 488/15

FSC 488/10

**4. Data Analysis Details**

**4.1 FCS Data File:** To request raw data please contact Dr. Chioma Okeoma.

**4.2 Compensation:** Not necessary since each fluorophore was used independently.

**4.3 Software:** Data was collected with FACSuite software (Becton, Dickinson and Company) and analyzed with FlowJo software (TreeStar).

**4.4 Gating Description**: After gating on the singlet bead population in FSC vs SSC plot, CD63 and CD9 expression was identified by histograms for the FITC and PE channels, respectively.
